# Supplementary figures and images for: scDIG: An R Shiny Application for Interactive Density-Based Gating of Single-Cell Proteomic and Transcriptomic Data
Source: bioRxiv. 2026 May 22:2026.05.20.726609. Preprint. [Version 1] doi: 10.64898/2026.05.20.726609 (PMC13228219; doi:10.64898/2026.05.20.726609)

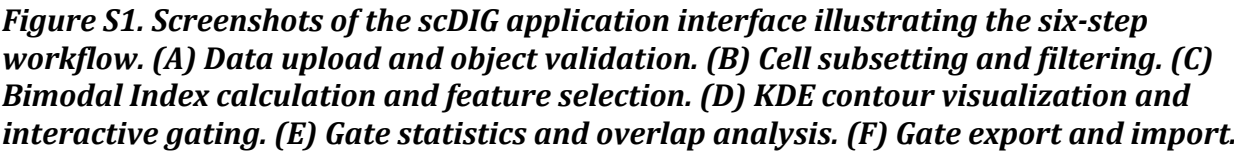

Supplement: 1 [file NIHPP2026.05.20.726609v1-supplement-1.pdf]
